# Single Cell Isolation Using Optical Tweezers

**DOI:** 10.3390/mi9090434

**Published:** 2018-08-29

**Authors:** Anusha Keloth, Owen Anderson, Donald Risbridger, Lynn Paterson

**Affiliations:** Institute of Biological Chemistry, Biophysics and Bioengineering, School of Engineering and Physical Sciences, Heriot Watt University, Edinburgh EH14 4AS, UK; anushakeloth89@gmail.com (A.K.); oa35@hw.ac.uk (O.A.); drr30@hw.ac.uk (D.R.)

**Keywords:** optical tweezers, optical trap, PDMS devices, single cells

## Abstract

Optical tweezers offer a non-contact method for selecting single cells and translocating them from one microenvironment to another. We have characterized the optical tweezing of yeast *S. cerevisiae* and can manipulate single cells at 0.41 ± 0.06 mm/s using a 26.8 ± 0.1 mW from a 785 nm diode laser. We have fabricated and tested three cell isolation devices; a micropipette, a PDMS chip and a laser machined fused silica chip and we have isolated yeast, single bacteria and cyanobacteria cells. The most effective isolation was achieved in PDMS chips, where single yeast cells were grown and observed for 18 h without contamination. The duration of budding in *S. cerevisiae* was not affected by the laser parameters used, but the time from tweezing until the first budding event began increased with increasing laser energy (laser power × time). Yeast cells tweezed using 25.0 ± 0.1 mW for 1 min were viable after isolation. We have constructed a micro-consortium of yeast cells, and a co-culture of yeast and bacteria, using optical tweezers in combination with the PDMS network of channels and isolation chambers, which may impact on both industrial biotechnology and understanding pathogen dynamics.

## 1. Introduction

In the last several years there has been a surge in attention towards single cell analysis due to increasing awareness of the importance of cell heterogeneity, advances in genome and transcriptome amplification and the emergence of technologies which enable single cell manipulation. Traditional ‘bulk’ studies on millions of cells in a single experiment can only provide general and averaged results regarding cell behaviour. However, even within a genetically identical population, cell heterogeneity exists, due to individual cells experiencing and reacting to differences in their micro-niche or due to stochastic gene expression. As such, single-cell isolation has become an important tool for researchers interested in purifying and analysing single cells to study cell heterogeneity [1] and subsequently investigating cell dynamics [2] or performing genome or transcriptome sequencing [3]. Single cell methods have become a key technique in prokaryotic biology as single cell isolation provides a means by which previously uncultured microbes can be grown in a lab by eliminating competition from faster growing organisms, or the link between microorganism and genome can reveal previously undiscovered microbial functions and metabolites from this ‘unculturable microbial dark matter’ [4]. Isolation of single cells is also key to reproduce a pure culture, where all cells in the culture are derived from a single progenitor cell [5]. Therefore, single cell technologies offer the ability to isolate a single cell from an interfering population and the study of individual cells, unbiased by population effects.

This paper summarizes state of the art, single cell isolation techniques and describes the use of optical tweezers to perform single cell isolation. Firstly we characterize our optical tweezers system by calculating the applied force on baker’s yeast, *S. cerevisiae*, cells. This is done by measuring the maximum velocity that the cells can be manipulated for a variety of laser powers and applying the velocity to the Stokes’ Drag equation using the appropriate corrections for our sample. We go on to use optical tweezers in combination with channel systems to physically separate individual cells from a community. Three channel systems for isolating cells are described and compared; a micropipette channel, a polydimethylsiloxane (PDMS) device and laser machined channels in fused silica. Biological cells used in these experiments are yeast, however bacteria and cyanobacteria are also isolated, demonstrating the wide applicability of optical tweezers and channel systems for single cell isolation. Finally, and significantly, the clonability of *S. cerevisiae* cells at three tweezing regimes used in isolation experiments is measured, to understand the effect that our optical tweezers system has on cell growth. We find that our optical tweezing parameters for single yeast cell manipulation enable viable cells to be quickly isolated, without the need for any microfluidic system, dynamic light pattern or image processing to be implemented, which has important implications given that precision isolation and cell viability are more highly ranked than throughput for many applications of cell isolation.

### 1.1. Single Cell Isolation Methods

In order to establish a pure culture, a viable cell must be isolated and this physical isolation must be maintained whilst the cell divides to form a colony. Similarly, in order to perform single cell ‘omics’, a cell must be physically isolated from other cells in the population. Cell isolation methods preferred by research groups depend on the nature of the sample (number of cells, origin of sample) and the processing to be performed on the isolated cells; culture-based or culture-independent analyses [6]. Isolation may be achieved by statistical means; by dilution to extinction whereupon a sample is diluted until, on average, there is only a single viable cell left in a given location, such as a well of a 96 well plate. It is simple and easy to perform, however there is no control over where each individual cell in the population goes and it does not necessarily provide single cells. 

Individual cells may be selectively isolated, rather than leaving the choice of cells to be investigated to chance, by using microscope-based techniques. Early techniques used micro-needles or microcapillaries connected to pressure and suction pumps to selectively micropipette individual cells and move them to another, sterile location, for example a microchamber [7,8]. The mechanical forces exerted on these cells are large, and can lead to shear damage, however, micromanipulation using hand-held or robotic micropipettes remains popular for cell isolation when working with small numbers of cells [6]. Laser capture microdissection (LCM) [9] is another isolation technique performed under a microscope, allowing a cell from a sample, spread on a sheet of thin polyethylene membrane, to be selected and cut-out using a laser. The laser beam circumscribes an area containing a cell of interest and the cut-out region falls, due to gravity into a microwell. Alternatively, the laser catapults the cut-out region into a microwell. Specimens were traditionally histopathological, so fixed in formalin, embedded in paraffin, or cryo-fixed but nowadays live cells can be isolated using LCM, as can prokaryotes [10] for downstream culture.

A popular method of cell isolation, aimed at sorting and analyzing large volumes of single cells in a short time, is fluorescence activated cell sorting (FACS) [11]. FACS systems can quantitatively analyze multiple characteristics of millions of single cells from a heterogeneous population and can be easily adapted to deflect a charged droplet containing a cell of interest into a microtiter plate. It can perform high-throughput single-cell analysis and isolate single cells of interest from thousands of cells in a population using up to 18 surface markers and can be used as a platform to select and isolate single cells for high-resolution Next Generation Sequencing analysis to resolve sample heterogeneity and reveal novel biology [12]. However, FACS systems typically require large sample sizes and are primarily designed to process eukaryotic cells and are not optimized for smaller microorganisms [13].

Compartmentalization techniques are also available and well suited for eukaryotic or prokaryotic cell isolation, such as ‘lobster traps’ which have been used to cage individual bacteria and investigate their growth and social dynamics [14,15]. Lobster traps are filled stochastically by flowing cells into them and hoping for one cell to enter the trap and proliferate in a confined volume. Microdroplet compartments can be created using a microfluidic network. They are similar to lobster traps since they provide a compartment in which single cells can be isolated, they are monodisperse and thus suitable for quantitative studies and in addition can be used for high-throughput experiments [16]. Typically, microdroplets are filled stochastically, but combining microdroplet generation methods with cell pre-selection is also possible, for example applying acoustic, optical or electric fields [17].

Active cell manipulation technologies, in which cells move in response to an applied field, have the advantage of being sterile, non-contact, manipulation methods capable of sorting populations of cells within microfluidic devices based upon the cells’ response to the field. Optical [18], acoustic [19,20] uniform and non-uniform electrical fields [21] and magnetic fields [22] all have uses in either label-free or labelled-cell sorting [23] and in some cases can be adapted for single cell isolation. 

Microfluidic or chip-based cell sorters have great potential for single cell isolation in microbiology and biotechnology because they can be used for a variety of sample sizes, including small, precious samples of few cells, or large samples which include interesting rare cells. They can offer precise isolation of selected cells, are disposable (thus reducing cross-contamination) and are potentially easier to use, smaller in scale and less expensive compared to some previously mentioned techniques. The use of optical tweezers in particular offers unparalleled selectivity of single cells, precision of translocation of a single cell, viability of isolated cells and potential for automation; desirable factors for many experiments requiring cell isolation.

### 1.2. Single Cell Manipulation and Isolation Using Optical Tweezers

Ashkin, the inventor of optical tweezers reported that optical trapping using an 80 mW laser beam at 1064 nm wavelength for 30 min did not affect growth or division of *E. coli* [24], however, absorption of laser light by a living cell may result in photodamage to the cell.

Work which followed Ashkin’s seminal paper reported negative effects of optical trapping including propagation ability [25,26,27] and cell motility [28], and the induction of a stress response gene in *C. elegans* [29]. In 2006 Ayano et al. found that *E. coli* cell growth stopped during optical trapping using a 1064 nm trap, even at very low trap powers. They found that damage to the cell’s growth and interdivision period was proportional to the total laser energy incident on the cell [30]. 

More recently, our group have measured localized heating in water by optical traps and found temperature increments of 99 °C/W for 980 nm laser light, 49 °C/W for 1090 nm, which are expected to cause photothermal damage to cells, and 0 °C/W for 808 nm and 750 nm light, all focused by a ×100, 1.3 NA objective lens [31]. Minimal photochemical damage occurs for laser wavelengths close to 820 or 980 nm as demonstrated by the cloning efficiency of mammalian Chinese Hamster Ovary (CHO) cells after irradiation with different wavelengths [26].

Despite these reports of photodamage to cells, optical tweezers, and optical forces in general have already been used successfully to manipulate single cells within a variety of devices for a range of applications, both in samples with flow and in static samples without flow. A dual beam tweezer was used in combination with an image processing algorithm to identify and isolate human peripheral blood cells based on their morphology, and erythrocytes were manipulated distances greater than 1 mm, for times longer than 20 min, without showing any morphologically visible photodamage [32]. 

Optical tweezers have been used to move single *E. coli* cells into individual chambers in a micro-chamber array. After a cell had divided, one of the two daughter cells was moved to a new chamber, allowing generational differences to be monitored. The adaptation of single cells to changes in nutrient concentration was observed for single *E. coli* tweezed into individual micro-chambers [33]. The ability to change the response of cells to changes in nutrient concentration is also important for studies of culturability. Optical tweezers have been combined with microfluidics to move *E. coli* cells between different reservoirs where they are exposed to different media containing fluorescent stains, without the media being dragged along with the trapped cells [34]. Single yeast cells have been optically manipulated in a nutrient gradient, created within a microfluidic device [35], thus cells are exposed to different environments and detection and analysis of rapid changes to the cells size in response to the osmolarity of the environment can be analyzed. The same group subsequently demonstrated rapid switching of the environmental glucose concentration around a yeast cell by combining microfluidics and optical tweezers, and observed the cycling of intracellular GPF tagged proteins between the nucleus and cytosol in response to the changes in glucose availability [36,37]. A simple DVD pickup has been adapted to tweezing colloid and red blood cells in a laminar flow to direct the cell to the correct output for isolation from the rest of the sample flow [38]. A dynamic optical tweezer has been used in combination with microfluidics and image processing to select out rare cells based on their morphology from a sample flow [39]. This was developed further to include microarrays into which cells docked. The arrays of cells could be simultaneously optically levitated and manipulated into a different microfluidic environment within the same chip [40]. More recently, Probst et al. demonstrated tweezing of single *E. coli* cells into cultivation compartments allowing individual cell selection and precision inoculation [41], unlike the stochastic flow-based filling of the compartments previously shown [15]. Cell growth was unaffected after irradiation using 1064 nm at 60 mW for under 1 min.

In this paper we demonstrate the isolation of single cells using optical tweezers in combination with three different cell isolation devices; a hollow glass microneedle channel, a PDMS-based device and a device inscribed in fused silica. In all cases the optical tweezer is a fixed light beam requiring no moving parts, the device contains a static sample requiring no microfluidic elements and cells are precisely translocated into new locations, physically separated from the rest of the sample population. Yeast cell growth after tweezing has also been quantified and compared with untweezed controls. The tweezing and isolation of single bacteria, cyanobacteria and yeast is achieved and the advantages and drawbacks of the three devices are discussed.

## 2. Materials and Methods 

An optical tweezer system operating at 785 nm was used to manipulate and isolate cells. LabVIEW (National Instruments, Berkshire, UK) was used to control stages, camera, image acquisitions and the laser. Three devices were fabricated and used in order to assess their suitability for cell isolation experiments. Cells used in experiments were primarily the yeast *S. cerevisiae*, however bacteria (*E. coli*, *B. subtilis*) and cyanobacteria cultures were also used.

### 2.1. Optical Tweezers System

**Figure 1:**
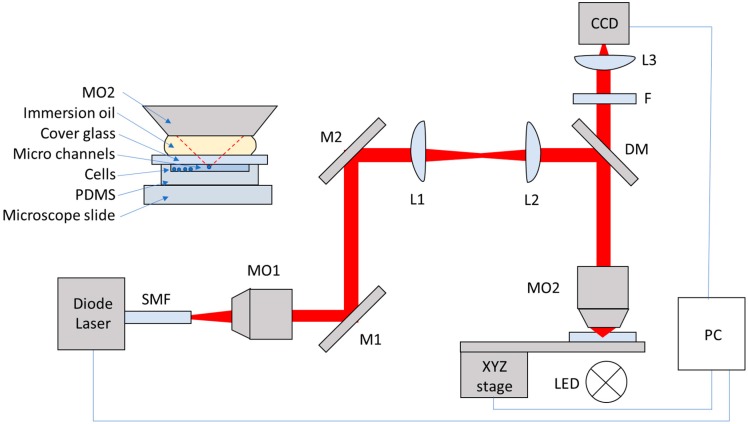
Optical tweezers. The beam emitted from the laser via a single mode fiber (SMF) is collimated by a 10× microscope lens (MO1). The two mirrors, M1 and M2, direct the laser light into an image relay system (L2 and L3). The laser light is reflected using a dichroic mirror (DM) into a ×100, 1.3 NA immersion objective lens (MO2). Inset is a sample which is placed on the sample stage.

A 785 nm laser diode (FPL785S-250, Thorlabs, Ely, UK) with maximum output of 250 mW and controlled via customized LabVIEW software using a controller (CLD1015, Thorlabs) was used in an optical tweezers assembly, as shown in Figure 1. The beam is directed via a dichroic mirror (DM) into the back aperture of a Nikon ×100, 1.3 NA objective lens (Thorlabs). A microscope stage is mounted on a Three-Axis Motorized Translation Stage (MT3/M-Z8, Thor labs). The stage is controlled via customized LabVIEW software using DC Servo Motor Controllers (KDC101, Thor labs). The sample is illuminated from below by an LED (MCWHL5, LEDD1B, Thorlabs) and imaged by a CCD camera (DCC1545M, Thorlabs).

### 2.2. Cell Tweezing, Imaging and Tracking

**Figure 2:**
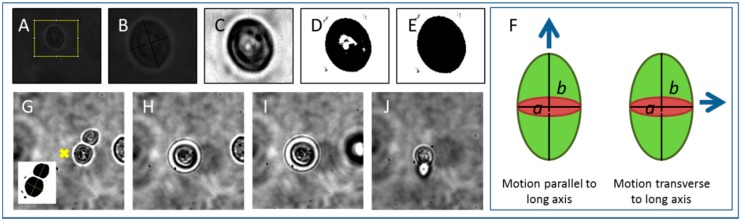
Yeast cell image analysis and tweezing. (**A**) Image of *S. cerevisiae* cell to be tweezed and its size analyzed; (**B**) zoom in using ImageJ; (**C**) enhanced contrast; (**D**) thresholding applied; (**E**) holes filled in, ‘particle’ to be measured in ImageJ. (**F**) Schematic of how cell is translated in optical tweezers; (**G**) *S. cerivisiae* with large daughter cell attached. Inset shows how cell dimensions, minor axis *a* and major axis *b* are measured in this case; (**H**) cell and attached daughter align in optical tweezer with long axis *b*, in direction of beam propagation; (**I**) cell and attached daughter are lifted in *z* using optical tweezers. A nearby cell is seen to move out of focus; (**J**) cell and attached daughter are tweezed at a velocity where they fall out of the trap.

Optical tweezers characterization was performed by selecting a single cell, translating it along the *z* axis by 40 ± 10 μm such that the cell is a distance away from the chamber surface and other cells, and measuring the critical velocity, $v_{c}$, at which the cell fell out of the trap when translated in the *x* direction. Cells were stably translated in the *x* direction a distance of 300 μm and back again at one velocity then the velocity was increased in 5 μm/s increments (using Thorlabs APT software) until the cell fell out the trap. This was repeated for approximately 20 cells at several laser powers. Cell images were captured using Thorcam software and processed using ImageJ (Figure 2A–E, Figure 2G inset). The cell minor (*a*) and major (*b*) axes were measured and images processed in ImageJ by background subtraction, contrast enhancement (Figure 2C), thresholding (Figure 2D), and filling in holes (Figure 2E) to generate a value for cell area.

The critical velocity, $v_{c},$ of cells at various laser powers is measured and used to measure Stokes’ drag force;

(1) $$F_{D}=6\pi r\eta v_{c}$$

to attain the trapping force, where *η* is the dynamic viscosity of water and *r* is the particle radius. Drag is known to increase if a particle approaches a wall of the channel, showing a linear relationship with *r*/*h* where *h* is the distance from the center of the particle to the coverglass [42,43]. Faxen provided a correction, *F_C_*, to the Stokes’ drag calculation to account for this wall effect for motion parallel to a flat wall (the type of motion in this paper) and is given by:

(2) $$F_{C}={\left[1-\frac{9}{16}\left(\frac{r}{h}\right)+\frac{1}{8}{\left(\frac{r}{h}\right)}^{3}-\frac{45}{256}{\left(\frac{r}{h}\right)}^{4}-\frac{1}{16}{\left(\frac{r}{h}\right)}^{5}\right]}^{-1}$$

It is worth noting here that a different correction exists for motion perpendicular to the wall [44].

Stokes’ Law addresses spherical objects, whereas *S. cerevisiae* (yeast) cells used here are prolate spheroid structures which reproduce asexually by developing a clone cell on their surface which eventually grows to the size of the parent cell and splits from it; an asymmetric division process known as budding. It is well known that non-spherical objects align with their long axis aligned to the axis of beam propagation in optical tweezers and indeed we observe this for individual yeast cells and cells with a budding daughter attached (Figure 2G–I and Video S1 in which a budding cell is tweezed using 1.8 mW through a series of higher velocities until it falls out at 0.01 mm/s). It is worth remarking here, that for nanoscale particles, elongated objects can be stably trapped with their long axis aligned along the polarization axis, transverse to the beam propagation axis, as shown by modelling a chain of two 100 nm spheres [45]. This may be of interest in the case of trapping ultramicrobacteria, for example rod-shaped mycoplasma. The axis of alignment is thought to change from the polarization axis to the beam propagation axis when an elongated particle is longer that 400 nm in length. The microbes discussed in this paper all have scales larger than this so align in the beam propagation axis.

In addition to Faxen’s correction, other corrections to the Stokes’ Drag for non-spherical objects have been proposed. The concept of an equivalent radius of an ellipsoidal particle could be used in place of radius in Stokes’ equation [43] or a correction may be used taking into account the aspect ratio for ellipsoidal and cylindrical particles, and *r*/*h* for particles oriented with their long axes parallel to the wall [46]. Corrections to Stokes drag for prolate spheroids, oblate spheroids and deformed prolate spheroids have been produced recently [47]. The correction for motion of the cell in the direction parallel to the long axis (as shown in Figure 2F, left) used in this work, from [43], is

(3) $$K_{a}^{\prime }=\frac{4}{3}\left(\beta^{2}-1\right)/\left(\frac{\left(2\beta^{2}-1\right)}{{\left(\beta^{2}-1\right)}^{1/2}}\right)ln\left[\beta +{\left(\beta^{2}-1\right)}^{1/2}\right]-\beta$$

where the aspect ratio $\beta =\frac{b}{a},$ as indicated in Figure 2F. This motion can be seen immediately before the cell falls out the trap in Video S1 and in Figure 2J. For motion transverse to the long axis of the cell (Figure 2F, right) the correction, from [43], is

(4) $$K_{b}^{\prime }=\frac{8}{3}\left(\beta^{2}-1\right)/\left(\frac{\left(2\beta^{2}-3\right)}{{\left(\beta^{2}-1\right)}^{1/2}}\right)ln\left[\beta +{\left(\beta^{2}-1\right)}^{1/2}\right]+\beta$$

which is the motion typically seen in a stably trapped *S. cerevisiae* cell translated in optical tweezers (Video S1, from which Figure 2G–J was taken). The forces with Faxen correction (*F_c_*) and ellipsoid corrections applied to Stokes’ drag force are shown in Table 1 in the results section for comparison.

**Figure 3:**
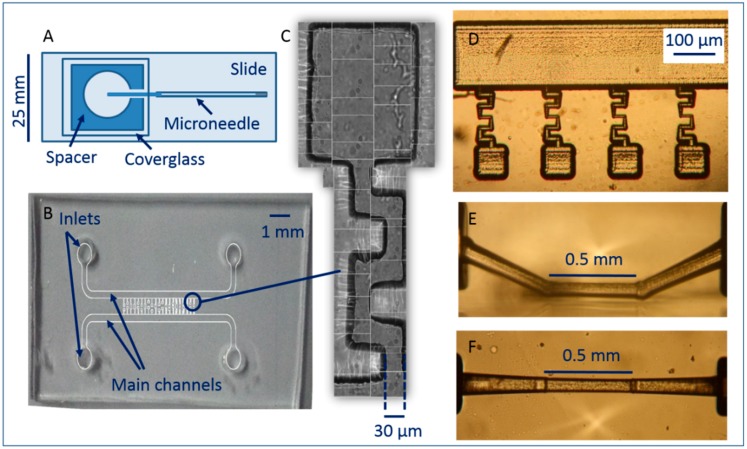
Chip designs. (**A**) Pulled micropipette-based chip; (**B**) PDMS chip; (**C**) channel and isolation chamber in PDMS chip; (**D**) ULI and chemically etched chip with channels on the surface of fused silica; (**E**) ULI and chemically etched sub-surface channel in fused silica, side view; (**F**) ULI and chemically etched sub-surface channel in fused silica, top view.

For tweezing experiments, LabVIEW was used to control laser power and on/off status, to translate the stage in 3D, to map the chips and set waypoints at the locations of chambers and isolated cells, to autofocus the microscope and finally, to capture time lapse images of isolated cells in their respective chambers. Experiments were performed at room temperature, 20 °C, except when isolating and growing *S. cerevisiae* which was at 30 °C. Initial work quantified the critical velocity that individual cells could be tweezed for different laser powers. This critical velocity and the measured cell dimensions were used to calculate Stokes’ Drag force. The corrections previously mentioned were also applied to generate an estimate for the force that our optical tweezers system could exert on cells. *S. cerevisiae* cells were tweezed and isolated in three types of device which are detailed in the following section. Following *S. cerevisiae* isolation, the isolation of different cell types, and co-cultures were attempted. Finally, as an indicator of phototoxic damage to the cell we have measured two features of the cell cycle. First, we measured the time taken for a daughter bud to become visible on cells which were optically tweezed. Second, we measured the duration of the budding event, from first observation of the bud on the surface of the mother cell until the daughter cell detaches. Time lapse imaging, controlled by LabVIEW, was used on multiple chambers in the PDMS device (Figure 3C) in which tweezed cells were located. Cells which had not been tweezed were also tracked as a control for adequate replication conditions, which were in water (nutrient-poor environment) and at 30 °C. An image of the cells was captured approximately every 13 min for 18 h.

### 2.3. Device Design and Fabrication

Three devices were made and tested in cell isolation experiments. Firstly, pulled, hollow, glass capillaries (also known as microneedles or micropipettes) were inserted into chambers to make a linear microchannel into which single cells could be optically manipulated and mechanically removed into a new sample (Figure 3A).

Secondly, a network of meandering channels was designed and fabricated using PDMS, a gas permeable elastomer (Figure 3B,C). This channel network was then bonded to glass to seal it and single selected cells were manipulated from a main channel, via the network, into vacant chambers. Finally, a network of channels was laser inscribed on the surface of fused silica (Figure 3D), loaded with sample, and then sealed with a coverglass and optical tweezing was performed within these channels. 

#### 2.3.1. Hollow Glass Microneedle Channel (Micropipette)

Microneedles were fabricated by pulling borosilicate capillaries (BF100-50-10, Sutter, Linton Instrumentation, Norfolk, UK) using a micropipette puller (P-97, Sutter) programmed to a temperature of 289 (RAMP), pull 30, velocity 120 and delay 200. These settings were optimal to produce hollow glass needles which had a narrow opening of tens of micrometers and a minimal taper over a length of over 1.5 cm. The microneedle was loaded via the unpulled end with sterile water, using a syringe and hypodermic needle and capillary sealant was used on the unpulled opening to prevent flow or evaporation in the microneedle. A sample chamber was made by placing an adhesive vinyl spacer (80 µm thick, 1.5 cm in diameter with a 1 cm hole and a notch cut out) onto a glass microscope slide. The chamber was filled with cells and a coverglass placed on top. The microneedle was directed under the coverglass, through the notch into the sample (Figure 3A). A drop of immersion oil was placed on the coverglass. The opening of the microneedle and surrounding cells were imaged under the ×100 objective lens in advance of optical tweezing.

#### 2.3.2. PDMS Chip

**Figure 4:**
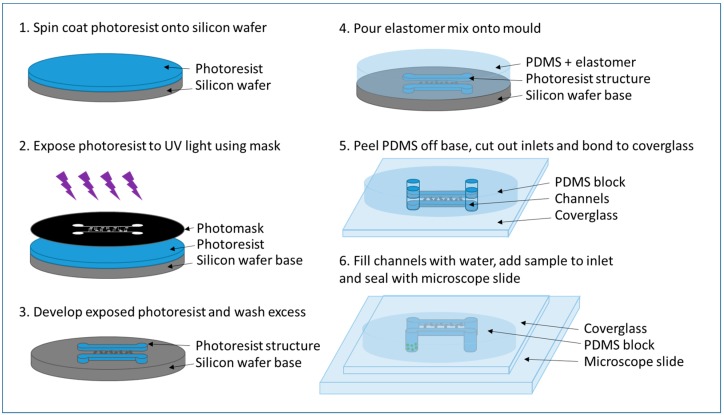
Soft lithography process for making PDMS devices.

Cell isolation chips were fabricated with polydimethyl siloxane (PDMS) on a microstructured mold. A schematic of the method is shown in Figure 4. PDMS is a polymer that can be easily molded into custom designs. Its optical transparency and gas-permeability make it particularly attractive for studying cells.

Patterns of channels and chambers required for cell isolation were generated using AutoCAD and transferred to a chromium (Cr)-on silicon glass plate mask with a resolution of 4 µm (by JDPhotodata, Hitchin, UK). This mask was used to create a relief of the chip structure using photoresist (AZ 2070, Microchemicals, Ulm, Germany) spin-coated at 750 RPM to a thickness of approximately 15 μm on a silicon wafer. The AZ 2070 substrate was soft-baked on a contact hotplate at 100 °C for 1 min and left overnight to evaporate the solvent and to increase the density of the film. The mask was placed on the wafer using a contact-aligner and the photoresist was exposed to UV light for one minute, followed by a post-exposure bake. Finally, the wafer was developed in AZ 726 MIF Developer (Microchemicals). The mold was placed in a close-fitting container and drop of Sigmacote was added offset from the channel structures. A lid was placed on the container and left for 30 min for the Sigmacote to evaporate, forming a hydrophobic layer on the mold. A 10:1 mixture of PDMS Sylgard 184 PDMS prepolymer and curing agent (Farnell, Leeds, UK) was made. This was poured onto the mold then dried overnight at room temperature to generate a negative relief of the photoresist mold. Once set, the PDMS block was cut from the mold using a scalpel and inlet and outlet holes were made in the block using a biopsy needle. The PDMS block was bonded to a clean coverglass by placing both block (channel side up) and coverglass in a reactive ion etcher (Plasmalab System100, Oxford Instruments, Abingdon, UK) and upon removal, pressing them together with a gentle force. They are stuck together with the channel structure facing towards the glass slide, taking care not to crush the channel structures. 

Once bonded, the chips were prepared by flushing the channels and chambers with water, media, or buffer via a syringe pump connected to a glass capillary inserted into one of the inlets. Finally, 2 µL of cells were injected into one inlet using a pipette. A microscope slide was placed on top to seal the device. A drop of immersion oil is placed over the channel structures on the coverglass side of the device and channels are inspected using a microscope to ensure chambers and channels are filled with water and ready for cell isolation experiments. 

The design of the structure comprises two main channels of width 350 µm, with 1 mm diameter inlets at either end. Along the length of these larger channels are a series of smaller (30 µm wide) meandering channels ending with an isolation chamber. The chamber dimensions depend on the type of cell to be isolated, and for yeast cells were designed to be 150 µm diameter (Figure 3C) with a volume of approximately 3.375 × 10^5^ µm^3^.

#### 2.3.3. Ultrafast Laser Inscription and Selective Chemical Etching of Cell Isolation Chambers

A third type of device in which to optically tweeze and isolate cells was made using channels and chambers made in fused silica. The chips were fabricated using the technique of ultrafast laser inscription (ULI) to write the channel structures in fused silica, followed by selective chemical etching of the modified structure, resulting in a surface network of channels and chambers on the fused silica, as seen in Figure 3D. This fabrication technique has been previously described [48,49]. The channels were filled with water by pipetting and a 2 µL cell sample was added at one end of the main channel. A coverglass was placed on top and a drop of immersion oil was added, above the channel structures. 

The technique of ULI offers a unique capability to write sub-surface microfluidics, so a sub-surface channel was also made using the same technique in order to quantify the ability to optically tweeze cells in buried channels. The channel can be seen in Figure 3E,F.

Of all the devices, the device shown in Figure 3A is the most straightforward to make. Pulled glass capillaries can be made in batches with several made after a few minutes. To make the full device takes thirty minutes maximum. The PDMS and ULI devices take substantially longer to make and prepare. Wafer preparation and photolithography is an overnight process, however a wafer can be reused numerous times and several devices can be written onto the wafer. In this work the PDMS mold is made in an overnight step, although this can be shortened by baking in an oven for an hour. It takes around 30 min to flush and prepare the device before use. The ULI process is an overnight laser writing step followed by a lengthy chemical etching step, taking from several hours to overnight depending on the complexity of the device. Despite the time to make a ULI device, they may be reusable after cleaning of the channels within the fused silica substrate, unlike the other two, disposable devices, and to the best of our knowledge have not yet been used for cell isolation. 

We perform some initial characterization of our optical tweezers system in a device similar to that shown in 3A but without the pulled microneedle, before moving on to using these three devices for cell isolation experiments.

## 3. Results and Discussion

### 3.1. Tweezing Characterization

**Figure 5:**
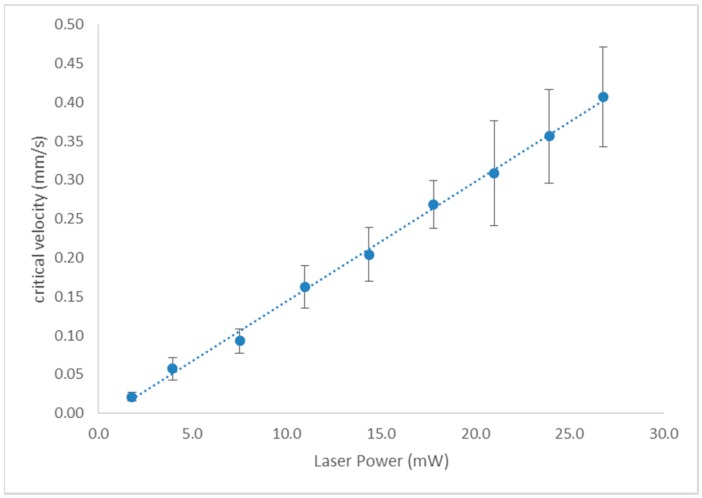
Dependence of critical velocity, *v_c_*, on laser power.

The maximum velocities for which 20 yeast cells could be tweezed were measured for nine laser powers to gauge the speed that cells could be manipulated through the isolation devices and also to estimate the force exerted on the cells. Data points show the average critical velocity of 20 cells and the error bars represent standard deviation. Experiments were performed in a device as shown in Figure 3A, between a glass microscope slide and a coverglass, separated by an 80 µm vinyl spacer, without the micropipette present. As expected, the maximum velocity a cell could be tweezed scaled linearly with laser power (Figure 5).

At a power of 1.8 ± 0.1 mW at the focal spot, the average maximum velocity a cell could be tweezed was 0.02 ± 0.01 mm/s, whereas at 26.8 ± 0.1 mW the average maximum velocity was 0.41 ± 0.06 mm/s, indicating that single cells may be rapidly selected and isolated using optical tweezers. Using higher laser tweezer powers of 300 mW from a 1064 nm Nd:YAG laser we note that yeast cells can be tweezed at approximately 2.5 mm/s. Forces used in this work have ranged from 1.04 ± 0.46 pN to 19.9 ± 5.8 pN, with laser powers of 1.8 mW to 26.8 mW, respectively, calculated using critical velocity, *v_c_* in Stokes’ Drag (Equation (1)) using the cell’s long axis, *b,* as the diameter. The error in the calculated force for each power arises from the uncertainty in the cell diameter of the 20 cells (the average diameter of 20 cells of different size is used for each power) and the uncertainly in the critical velocity, as the average maximum velocity of 20 cells is used. Faxen’s correction (Equation (2)) and the ellipsoidal shape corrections to Stokes’ Drag Force (Equations (3) and (4)) result in negligible changes as shown in Table 1, primarily because the aspect ratio of yeast cells is not usually greater than two except when a daughter cell is in the process of budding, and only single, non-budding cells were used here. In addition, the tweezed cells are lifted using the optical tweezer to the center of the channel so are at a distance of at least four times the cell diameter away from a wall. The main difference between the calculated forces is due to the use of either the length or width of the cell as the diameter with which to calculate Stokes’ Drag Force.

**Figure 6:**
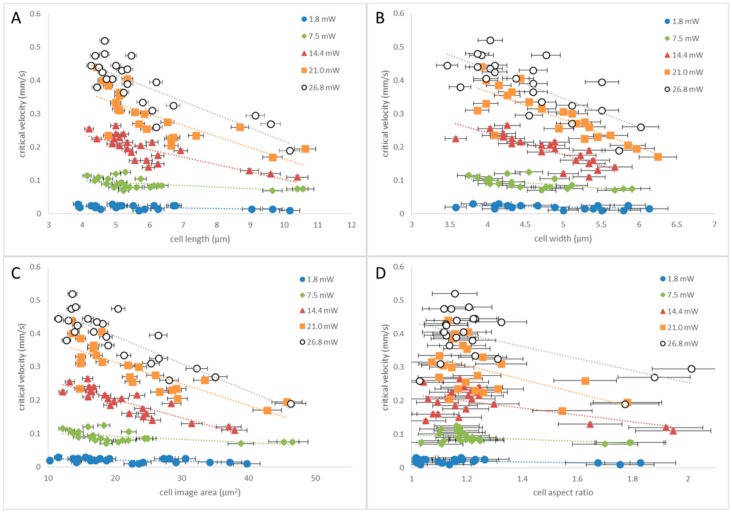
Critical velocities, *v_c_* plotted against yeast cell dimensions. (**A**) Critical velocity (*v_c_*) vs. cell length (*b*); (**B**) *v_c_* vs. cell width (*a*); (**C**) *v_c_* vs. cell area; (**D**) *v_c_* vs. aspect ratio (*b/a*). Lines act as a guide to the eye.

We have plotted critical velocities of cells for a variety of laser powers against cell length (*b*), cell width (*a*), cell area (measured in ImageJ) and aspect ratio (*b*/*a*) (Figure 6A–D respectively) and see that all four cell parameters scale with critical velocity and that shorter, narrower cells with aspect ratios closer to one can be tweezed with larger velocity. Twenty single cells, of different dimensions but with no visible sign of a budding daughter cell, and three dividing cells have been measured for each laser power. The three larger, dividing cells for each power can be seen as the data points with longer length, larger area and larger aspect ratio in Figure 6A,C,D respectively. The uncertainty in critical velocity arises from the incremental measurement in cell velocity of 5 μm/s and the uncertainty in dimension (3% for length, 4% for width and 5% for area) comes from comparing captured images with ImageJ which were either in focus or slightly out of focus.

Figure 6 shows that as *b*, *a*, area and aspect ratio of a cell increases, the maximum velocity that a cell can be tweezed decreases. Unsurprisingly, cells with a large, attached, daughter cell have an aspect ratio greater than 1.5 due to their longer length *b* compared to single cells, but similar sized minor axis *a* and as such are more difficult to tweeze than single cells. Having characterized the optical tweezers system and observed that yeast cells can be manipulated at velocities of several hundred micrometers per second at modest laser powers we now go on to perform some isolation experiments within different channel systems and apply the optical tweezers to some different cell types.

### 3.2. Tweezing in Isolation Devices

In an initial experiment to observe the difference in velocity that yeast cells could be trapped within the different devices, the maximum velocity, *v_c,_* recorded at a power of 40 mW (at the limit of our system and not used for isolation) was measured. In the device shown in Figure 3A an *S. cerevisiae* cell could be manipulated at a maximum velocity of 0.77 ± 0.01 mm/s. The depth of this sample is 80 µm and cells are lifted 40 ± 10 μm from the bottom, thus well away from any boundary. At the same power, within the main channel of a PDMS chip (such as that shown in Figure 3B) the maximum velocity was measured to be 0.24 ± 0.01 mm/s. The channel height in these devices is only 15 µm so the lower maximum speed is due to the closeness of the cell and a wall. The PDMS surface may undulate, knocking the cell out of the trap as it is translated, or the trapped cell may occasionally bump into an untrapped cell. This could be avoided by making deeper and wider channels in future. If either the top or bottom surface of the PDMS chip is not flat, this will lead to the focal position of the trap drifting in *z* as the device is translated in *x* or *y*. The focal spot will acquire spherical aberrations if its *z* position changes and the trap will become unstable. Care must be taken in the device fabrication process to ensure flat surfaces. In the ULI sub-surface chip (as seen in Figure 3E,F) the maximum velocity was 0.37 ± 0.01 mm/s. In this case the cell was translated in a channel 100 μm wide and deep, so could be kept well away from any surface, however the roughness of the etched glass at the top of the channel through which the tweezing beam must be directed is visible in Figure 3F, and results in beam aberration. This could be mitigated by annealing the channels after etching, to smooth the rough structures.

Having made some initial observations on the ability to tweeze yeast cells in the different devices, we next performed yeast cell isolation.

### 3.3. S. cerevisiae Isolation

**Figure 7:**
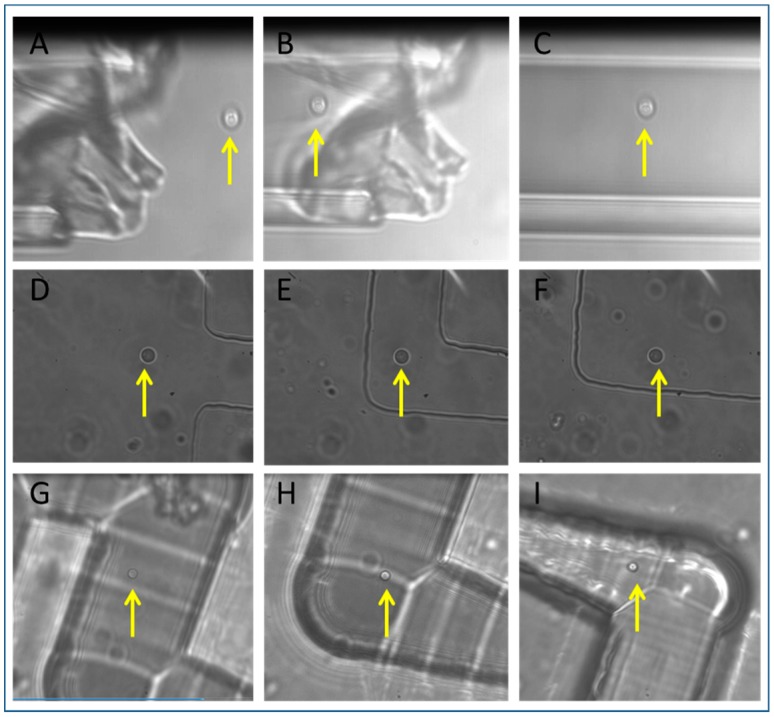
Tweezing and isolation of *S. cerevisiae* in 3 devices. (**A**) Micropipette, (**B**) PDMS chip, (**C**) ULI chip. The diameter of yeast cells (highlighted by yellow arrow) is approximately 5 µm.

In the chip shown in Figure 3A, cells were relatively easily prepared and isolated by manipulating the cell into a pulled glass micropipette (Figure 7A–C). A cell was found under the microscope, and manipulated towards the micropipette tip at velocities of several hundred μm/s, and then once the tip was located the cells were moved carefully at μm/s to maneuver the cell through the channel opening. Once within the channel, the cell could again be translated at hundreds of μm/s and be transported millimeters along the channel away from the tip. 

Removing the micropipette from the device and ensuring that the isolated cell could be used to re-seed a new culture was problematic, so although isolation from the surrounding population was achieved, the extraction of a single *S .cerevisiae* was not achieved in this case. Single yeast cells were also isolated in the PDMS device (Figure 7D–F). A cell was selected in the main channel (Figure 7D), tweezed at tens to hundreds of μm/s to the entrance of a meandering channel and then maneuvered though the meandering channel at a lower speed (Figure 7E) and finally deposited in the isolation chamber (Figure 7F, Video S2). Upon cell isolation in the PDMS chip, observation of the chamber over two days did not reveal any flow or movement of other cells into the chamber. A noticeable loss of liquid was observed after three days, and the chamber dried out. This needs to be taken into consideration if one intends to observe the dynamics of microorganisms for periods longer than three days. Preparing the cell sample in the ultrafast laser inscribed, chemically etched isolation device, shown in Figure 3D, was problematic as the channels were written on the surface of fused silica, not sub-surface, and a coverglass was placed on top after loading with sample. The coverglass displaced the sample such that it was difficult to contain the cells in the main channel and prevent them from entering the meandering channels. Isolation of a single yeast cell was achieved only after many attempts (Figure 7G–I), however fabricating a more complex design, similar to Figure 3D in a sub-surface chip with inlets and outlets (as Figure 3E,F shows) may lead to a more readily contained sample. A point to note here is that, unlike PDMS, fused silica is not gas permeable, so isolation chips made in this way will facilitate studies with obligate anaerobic microorganisms where absence of oxygen is required in the microenvironment. Very few studies have been published using these organisms, but they are abundant in nature and important for medical and industrial processes [50]. Having isolated yeast cells in all three devices, we went on to explore isolation and co-cultures using other cell types.

### 3.4. Isolation of Other Cell Types

As mentioned above, in the micropipette channel device shown in Figure 3A, removing the micropipette from the device and ensuring that the isolated cell could be used to re-seed a new culture was problematic, however, this was achieved with single cyanobacteria (Video S3), of similar dimensions to yeast. The contents of the micropipette were dispensed into a microfuge tube and left for three months to grow. They are extremely slow growing organisms, so clonability studies were not performed. The original sample of cyanobacteria was environmental so had other bacteria present. Although a culture of cyanobacteria grew from the isolated cell, it was contaminated with environmental bacteria. This means that a second step to the isolation process may be required to remove the contaminants, such as UV exposure, which may be detrimental to the cells of interest, or by tweezing out individual contaminants as soon as they are observed. Occasionally flow was observed in the micropipette and when removing from the device, relatively large mechanical forces would exacerbate this flow. Single bacteria (*B. subtilis*) were also isolated into a micropipette using this method (Video S4), and again, large mechanical forces experienced by the micropipette upon removal made it problematic to extract a single cell and re-seed a pure culture.

In addition to yeast cells, bacteria were also isolated in the PDMS device shown in Figure 3B. Bacteria were loaded into the main channel via an inlet and tweezed into the isolation chamber (Video S5 shows two *B. subtilis* cells isolated together).

**Figure 8:**
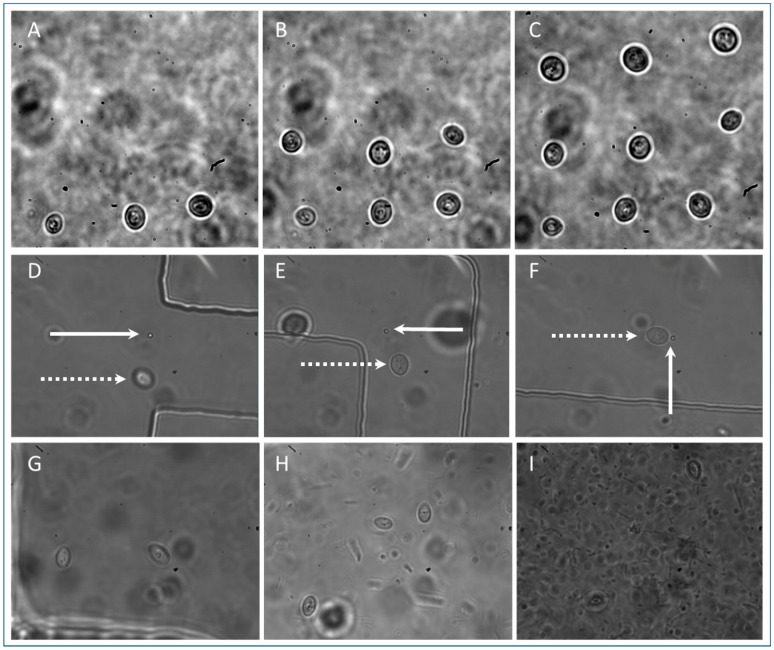
Setting up co-culture, (**A**–**C**) the creation of a 3 × 3 array of yeast cells; (**D**–**F**) a single *S. cerevisiae* yeast (dashed arrow) and single *E. coli* bacteria (solid arrow) tweezed through meandering channel in PDMS device into isolation chamber; (**G**) two yeast cells deposited by optical tweezers into PDMS isolation chamber at *t* = 0; (**H**) *t* = 1 day showing three yeast cells and bacterial contamination; (**I**) *t* = 5 days, two yeast cells are visible, the third is at the edge of the chamber and bacteria are numerous.

From these studies, PDMS chips proved the most successful of the three devices to work with in combination with optical tweezers for single cell isolation. To further demonstrate the applicability to single cell studies, and the study of dynamics of small numbers of cells, arrays of cells were created. Figure 8A–C shows stages of the creation of a 3 × 3 array of yeast cells. 

The average diameter of the cells is 5 µm and they are spaced approximately 10 µm apart. Each cell was exposed to 25 mW of laser power for tens of seconds. Time lapse imaging shows that in this environment (water, 30 °C) they do not drift out of the 50 µm field of view for at least 15 min (Video S6, one frame/30 s). A more viscous environment may be used to prevent drifting, the chambers may be coated to enable cells to stick in place, or a smaller chamber may be used. To fix cells in position a spatial light modulator (SLM) may be used to create a pattern of traps in 2D or 3D and the surrounding media may be polymerized [51]. This yeast array is constructed from cells of the same population, however co-cultures of cells from different populations may also be created. A single yeast cell and a single *E. coli* bacterium have been tweezed together into an isolation chamber to demonstrate that different cell types may be loaded into the device to establish a co-culture (Figure 8D–F). Bacteria were pipetted into one of the inlets (in Figure 3B) and yeast were pipetted into a second inlet. A single cell of each type is brought to the opening of a meandering channel (Figure 8D) and both are transported into an isolation chamber (Figure 8F). This has great potential for setting up microbial consortia to study the effects of one cell type upon another cell type using minimal reagents, space and equipment, and will impact on the fields of synthetic biology and industrial biotechnology [52] and understanding disease [2].

Sterility is an important factor as Figure 8G–I shows contamination of a yeast cell co-culture of two cells by an unidentified bacteria. There is no indication that there is contamination on day zero (Figure 8G) when the co-culture is established, but by day 5 (Figure 8I) the bacteria are numerous and yeast dynamics can no longer be observed. Microbiological aseptic technique was not adhered to in this case but should be implemented in future experiments to avoid contamination of cultures.

Now that cell isolation has been achieved, we now go on to quantify yeast cell doubling time after tweezing as an indicator of phototoxic damage to cells. We compare budding of cells tweezed under three regimes (25.0 ± 0.1 mW for 30 s, 25 ± 0.1 mW for 1 min, 19 ± 0.1 mW for 1 min) and compare with control cells which have not been exposed to the tweezing laser. We focus on *S. cerevisiae* as their cell cycle durations are easy to measure by the budding of a daughter cell and cells are relatively static so can be tracked using time lapse imaging. Other cell types isolated have not been measured in this way as bacteria are too mobile to track with our current time lapse imaging software and *cyanobacteria* take weeks to divide, which is again difficult to track with our current monitoring software.

### 3.5. S. cerevisiae Doubling Time

**Figure 9:**
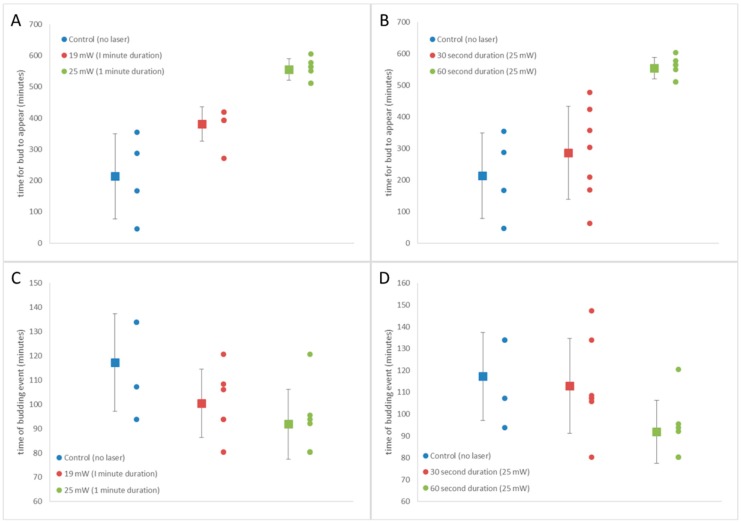
(**A**) Time for *S. cerevisiae* bud to appear on mother cell surface after tweezing for three laser powers (0, 19 and 25 mW). (**B**) Time for bud to appear for three 25 mW laser durations (0 s, 30 s and 60 s), (**C**) The duration of budding events on single cells, from first observation of bud on surface of mother cell until daughter cell detached from mother cell, for three laser powers (0, 19 and 25 mW), (**D**) duration of budding event for cell exposed to 25 mW laser of different duration (0, 30 and 60 s).

The laser power used and duration a cell is exposed to the tweezing beam dictates the total energy incident upon the particle, which contributes to the extent of both photothermal and photochemical damage sustained. It is known that shock responses in cells can lead to changes in characteristic cell cycle features because they transiently inhibit the mechanisms of cell division and it has also been considered that optical trapping prevents free movement of molecules which play a dynamic role in cell division [30]. As a measure of damage to the cell by the tweezing laser we have plotted the time it takes a small number of tweezed cells to begin budding, compared to untweezed controls which have not been exposed to any laser radiation (Figure 9A,B). We have also plotted the duration of the budding event, defined as the time from when a bud is first observed in the time lapse images until the time in which the daughter cell separates from the mother cell, for tweezed cells and untweezed controls (Figure 9C,D). Cells were not synchronized, so single cells of similar size with no visible bud were selected for tweezing. 

**Figure 10:**
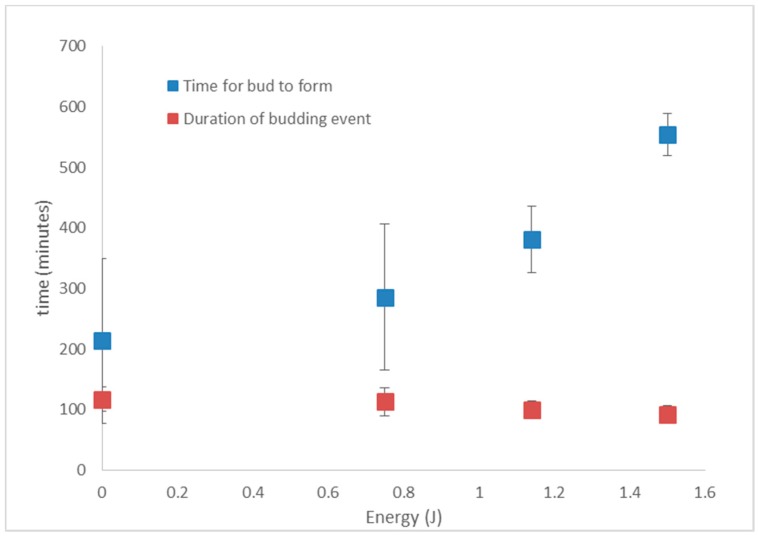
The dependence of time for bud to appear on tweezed cell, and the duration of the budding event on the laser energy.

Figure 9A,C shows results for cells exposed to no laser beam (Control *n* = 4), cells exposed to 19 mW for 60 s (*n* = 6) and cells exposed to 25 mW for 60 s (*n* = 7). The average time to bud after tweezing, with error bars showing standard deviation, is plotted to the left of the data points in Figure 9A, and similarly for the average time of the budding event in Figure 9C. The time for budding to occur in the asynchronous yeast cells that were not tweezed (control (no laser)) was on average 213 ± 136 min. Even with the small number of cells measured, this is comparable to 207 min measured by Leitao and Kellog [53] for yeast cells growing in a nutrient-poor environment. Cells tweezed at 19 mW have a longer average time until budding of 381 ± 55 min and cells tweezed at 25 mW for the same duration of 60 s have a longer time of 554 ± 34 min until the appearance of a bud. The duration of the budding event, measured from when the bud first appears until when the daughter cell detached from the mother (Figure 9C) in untweezed control cells was 117 ± 20 min (*n* = 4) and again this is similar to Leitao and Kellog’s measurement of 60 min for cells in a nutrient-poor medium. We note that they measure the duration of metaphase and anaphase of the cell cycle, whereas we measure the full budding event which begins in G2 and ends with cytokinesis, hence our measured time is longer. There is not any significant difference in the duration of the budding event between the control cells, and those exposed to 19 mW (100 min, *n* = 6) and 25 mW (92 min, *n* = 7) (Figure 9C). When the exposure time to the laser beam is zero, 30 s (at 25 mW) or 60 s (also at 25 mW) we see an increase in the time taken for the cell to bud with an increase in exposure time (Figure 9B). As already mentioned, it takes on average of 213 ± 136 min for the control cells to produce a bud, 286 ± 148 min for the cells exposed for 30 s (*n* = 7) and 554 ± 34 min for the cells exposed to 60 s (*n* = 7) to produce an observable bud. Increasing the duration of laser exposure does not significantly impact on the duration of the budding event (Figure 9D), with control, 30 and 60 s taking an average time of 117 ± 20 min (*n* = 4), 113 ± 22 min (*n* = 7) and 92 ± 14 min (*n* = 7). Recent work by Pilat et al. has shown that tweezing *S. cerivisiae* in a 1064 nm optical trap with 19 mW of laser power for 15 min resulted in no delay of reproduction although it did reduce the mean cell size [52]. We did not measure cell size in this experiment so cannot comment on how our tweezing parameters affect cell size. Pilat et al. also showed that at 38 mW there was a significant delay in reproduction and at powers above 76 mW half of all tweezed cells died. Taking into account the energy deposited on the cell (laser power x trapping time), the time taken for the bud to appear was plotted, as was the duration of the budding event against energy (Figure 10).

There is no notable difference in the budding time for the laser parameters tested. With further study it may become evident that the size of the daughter bud (or the growth rate of the daughter cell) is affected. The time for a bud to appear after tweezing does increase in an energy dependent manner, with buds taking longer to appear if the mother cell was exposed to a higher laser energy. At 1.5 J this became particularly evident. Ayano et al. found, when tweezing *E. coli* with 1064 nm, cell division activity was normal when the total energy was less than 0.36 J, whereas 1.06 J of total energy stopped cell division [30]. Recent work [54] found *S. cerevisiae* in a nutrient-rich environment tolerant to laser powers of 38 mW for 15 min (34 J), where 7% of cells died and trapped cells showed a less than 20% increase in the time between the first bud and the second bud appearing on the mother cell (114 min compared to 135 min). The delay in bud appearance observed here is likely to be induced by a checkpoint- induced delay which slows the cell cycle to allow correction of aberrant DNA structures and incomplete kinetochore assembly and thus protects genomic integrity.

## 4. Conclusions

Optical tweezers offer unparalleled selectivity of single cells, precision of translocation of a single cell, viability of isolated cells and potential for automation; desirable factors for many experiments requiring cell isolation. Whilst higher powers will result in faster tweezing and isolation, a power of around 25 mW at 785 nm, exposed to a cell for one minute, is sufficient for isolation and subsequent division of yeast cells. At this power, single cells can be manipulated at 200–300 μm/s, ample for maneuvering them with isolation devices, such as those presented. 

Cells are readily prepared and isolated using optical tweezers in combination with PDMS devices consisting of the design shown in Figure 3B,C, whereas glass microneedle channels and laser written channels on the surface of fused silica posed challenges for sample preparation and isolating single cells from the population. PDMS and ULI chips have the potential to integrate microfluidics if necessary, in which case cell perfusion may be controlled in a manner to interrupt or induce cell growth using selected media. By integrating microfluidics a single cell can be subject to many microenvironments without being removed from the field of view of the microscope and subsequent dynamics can be studied in real time. These devices are also amenable to functionalization with heaters, electrodes and sensors, enabling a host of studies.

Considering that for many users and applications cell viability is ranked higher than throughput when desirable benefits of single cell isolation technologies are listed [6] the full potential of optical tweezers remains to be realized in this field.

## Figures and Tables

**Table 1:** Stokes’ Drag Forces with no correction applied (*F_D_*) (Equation (1)) and with Faxen’s correction (*F_c_*) (Equation (2)) and ellipsoidal shape correction ($K_{a}^{\prime }$ (Equation (3)) and $K_{b}^{\prime }$ (Equation (4)) applied. *a* = minor axis (cell width), *b* = major axis (cell length) and *d* = particle diameter.

Correction Applied Power (mW)	$F_{D}\;(b=d)\;\mathrm{No}\;\mathrm{Correction}$ (N)	$F_{D}\;(a=d)\;\mathrm{No}\;\mathrm{Correction}$ (N)	$F_{C}(b=d)$ (N)	$F_{C}\;(a=d)$ (N)	$K_{a}^{\prime }\;(a=d)$ (N)	$K_{b}^{\prime }\;(b=d)$ (N)
1.8 ± 0.1	1.04 ± 0.46 × 10^−12^	1.88 ± 0.83 × 10^−12^	1.08 ± 0.48 × 10^−12^	1.94 ± 0.85 × 10^−12^	1.92 ± 0.84 × 10^−12^	1.09 ± 0.48 × 10^−12^
4.0 ± 0.1	2.87 ± 1.08 × 10^−12^	4.98 ± 1.87 × 10^−12^	2.98 ± 1.12 × 10^−12^	5.15 ± 1.93 × 10^−12^	5.13 ± 1.92 × 10^−12^	3.04 ± 1.14 × 10^−12^
7.5 ± 0.1	4.61 ± 1.32 × 10^−12^	7.87 ± 2.25 × 10^−12^	4.79 ± 1.37 × 10^−12^	8.13 ± 2.33 × 10^−12^	8.14 ± 2.33 × 10^−12^	4.93 ± 1.41 × 10^−12^
11.0 ± 0.1	8.12 ± 2.27 × 10^−12^	1.39 ± 0.39 × 10^−11^	8.43 ± 2.36 × 10^−12^	1.44 ± 0.40 × 10^−11^	1.44 ± 0.40 × 10^−11^	8.66 ± 2.43 × 10^−12^
14.4 ± 0.1	1.05 ± 0.31 × 10^−11^	1.81 ± 0.53 × 10^−11^	1.09 ± 0.32 × 10^−11^	1.87 ± 0.55 × 10^−11^	1.86 ± 0.55 × 10^−11^	1.11 ± 0.33 × 10^−11^
17.8 ± 0.1	1.35 ± 0.38 × 10^−11^	2.3 ± 0.58 × 10^−11^	1.4 ± 0.35 × 10^−11^	2.38 ± 0.60 × 10^−11^	2.38 ± 0.60 × 10^−11^	1.44 ± 0.36 × 10^−11^
21.0 ± 0.1	1.62 ± 0.60 × 10^−11^	2.74 ± 1.01 × 10^−11^	1.68 ± 0.62 × 10^−11^	2.83 ± 1.04 × 10^−11^	2.84 ± 1.05 × 10^−11^	1.74 ± 0.64 × 10^−11^
23.9 ± 0.1	1.77 ± 0.53 × 10^−11^	3.07 ± 0.92 × 10^−11^	1.84 ± 0.55 × 10^−11^	3.18 ± 0.96 × 10^−11^	3.17 ± 0.95 × 10^−11^	1.88 ± 0.57 × 10^−11^
26.8 ± 0.1	1.99 ± 0.58 × 10^−11^	3.41 ± 1.00 × 10^−11^	2.06 ± 0.61 × 10^−11^	3.52 ± 1.04 × 10^−11^	3.52 ± 1.04 × 10^−11^	2.12 ± 0.62 × 10^−11^

## Supplementary Materials

The following are available online at http://www.mdpi.com/2072-666X/9/9/434/s1, Video S1: Budding yeast cell critical velocity measurement, Video S2: Yeast tweezing in PDMS device, Video S3: Cyano tweezing in micropipette, Video S4: B.Subtilis tweezing in micropipette Video S5: Two bacteria tweezing in PDMS, Video S6: Yeast 3 × 3 array dynamics (1 frame per 30 s).
